# Transcriptional changes in response to X chromosome dosage in the mouse: implications for X inactivation and the molecular basis of Turner Syndrome

**DOI:** 10.1186/1471-2164-11-82

**Published:** 2010-02-01

**Authors:** Alexandra M Lopes, Paul S Burgoyne, Andrew Ojarikre, Julien Bauer, Carole A Sargent, António Amorim, Nabeel A Affara

**Affiliations:** 1 IPATIMUP, Instituto de Patologia e Imunologia Molecular da Universidade do Porto, R Dr Roberto Frias S/N, 4200-465 Porto, Portugal; 2 Department of Pathology, University of Cambridge, Tennis Court Road, Cambridge, CB2 1QP, UK; 3 Division of Stem Cell Biology and Developmental Genetics, MRC National Institute for Medical Research, London, NW7 1AA, UK; 4 Faculdade de Ciências, Universidade do Porto, 4099-002 Porto, Portugal

## Abstract

**Background:**

X monosomic mice (39,XO) have a remarkably mild phenotype when compared to women with Turner syndrome (45,XO). The generally accepted hypothesis to explain this discrepancy is that the number of genes on the mouse X chromosome which escape X inactivation, and thus are expressed at higher levels in females, is very small. However this hypothesis has never been tested and only a small number of genes have been assayed for their X-inactivation status in the mouse. We performed a global expression analysis in four somatic tissues (brain, liver, kidney and muscle) of adult 40,XX and 39,XO mice using the Illumina Mouse WG-6 v1_1 Expression BeadChip and an extensive validation by quantitative real time PCR, in order to identify which genes are expressed from both X chromosomes.

**Results:**

We identified several genes on the X chromosome which are overexpressed in XX females, including those previously reported as escaping X inactivation, as well as new candidates. However, the results obtained by microarray and qPCR were not fully concordant, illustrating the difficulty in ascertaining modest fold changes, such as those expected for genes escaping X inactivation. Remarkably, considerable variation was observed between tissues, suggesting that inactivation patterns may be tissue-dependent. Our analysis also exposed several autosomal genes involved in mitochondrial metabolism and in protein translation which are differentially expressed between XX and XO mice, revealing secondary transcriptional changes to the alteration in X chromosome dosage.

**Conclusions:**

Our results support the prediction that the mouse inactive X chromosome is largely silent, while providing a list of the genes potentially escaping X inactivation in rodents. Although the lower expression of X-linked genes in XO mice may not be relevant in the particular tissues/systems which are affected in human X chromosome monosomy, genes deregulated in XO mice are good candidates for further study in an involvement in Turner Syndrome phenotype.

## Background

The existence of dimorphic sex chromosomes poses a challenge to the balance of gene dosage between the sexes. In mammals, X chromosome inactivation is the mechanism by which the equality in gene expression between males and females is restored, through the transcriptional silencing of one of the X chromosomes in females. Dosage compensation between X-linked and autosomal genes is also achieved, through the upregulation of the active X chromosome in females and the single X chromosome in males [1].

In eutherian mammals, one X chromosome is randomly inactivated in the cell lineages of the embryo proper, early in development. The initiation of X-inactivation is controlled by a region on the X chromosome, the X-Inactivation Centre (*XIC *in humans and *Xic *in mice), which contains the X-Inactive-Specific Transcript locus (*XIST/Xist*), a noncoding RNA essential for silencing, and several other genetic elements (reviewed by [2]). The nature of their interactions and the succession of events that results in the global repression of X-linked genes are not fully understood, being the focus of active research [3,4]. Once initiated, a wave of silencing spreads through the entire chromosome and the transcriptionally silent state is stably maintained on the inactive X chromosome by various epigenetic modifications, such as histone modifications and DNA methylation [2,5] and inherited clonally in subsequent cell divisions.

Although X inactivation is a very effective mechanism some genes are expressed from the inactive X chromosome [6]. Using *in vitro *models Carrel and Willard [7] performed a systematic analysis of human X-linked transcripts and predicted that approximately 15% of the genes on the human X chromosome escape X inactivation to variable extents. In the mouse only a few "escapees" have been reported [8-12] and the finding of differences in the inactivation status of several genes in a conserved region between the human and mouse X chromosomes has been taken as evidence that the mouse X chromosome is depleted of genes escaping X inactivation [13]. Although the mechanisms of escape are still poorly understood, from the analysis of sequence features within the relevant regions in human and in the mouse, the authors suggested that both genomic context and gene-specific regulatory elements are involved. In particular, long terminal repeats (LTRs) are more frequent in a smaller human X chromosome domain where all genes escape inactivation, compared to the syntenic region in the mouse, where only one of the genes, *Jarid1c*, is expressed from the inactive X [13]. Additionally, several binding sites for the insulator protein CTCF were identified in the 5' end of *Jarid1c *but not in its human homologue (JARID1C), raising the hypothesis that this protein may be required to prevent the spreading of silencing epigenetic marks to *Jarid1c*, which is embedded in inactive chromatin on the mouse X chromosome [14]. Recently, the analysis in female ES cells of chromatin modifications accompanying XCI has revealed an increase of H3K27me3 throughout the inactive X chromosome, as inactivation proceeds, and a preferential localisation of this silencing mark over active genes [15]. The characterization of a larger number of genes expressed from the inactive X chromosome in the two species would certainly contribute to a better understanding of the process of escape, but a comprehensive search for genes escaping inactivation on the mouse X chromosome is still lacking.

In recent reports, microarray technology was used to compare gene expression between males and females in several mouse tissues, from which indirect evidence of escape from X inactivation can be derived [1,16,17]. Nevertheless in these analyses it is difficult to disentangle the effects of sex hormones in the regulation of gene expression and, as later demonstrated using manipulated mice [18], these are indeed substantial. In the mouse model used to demonstrate this, the gonadal phenotype (ovaries or testis) is independent of the sex chromosome complement (XX or XY) and therefore it circumvents the confounding effects of differential hormonal status. This strategy had been previously used by Xu and collaborators to investigate expression levels of several sex-linked genes in brain and it was demonstrated that *Eif2s3x, Jarid1c, Utx *and *Usp9x *were more highly expressed in the XX genotype, compared to XY, regardless of the gonadal phenotype [19-23], which further suggests that these genes escape X inactivation in the mouse.

The 39,XO mouse is another valuable model to assess the impact of the number of X chromosomes on the level of X-linked gene expression in a similar hormonal milieu, using a simple experimental design. The underlying assumption is that genes that are expressed from both the active and inactive X chromosomes would be expressed at higher levels in the XX females than in their XO littermates. We generated three genotypes (X^m^O, X^p^O and X^m^X^p^) using the breeding strategy of Davies *et al*.[24]. The XO mice are free from cryptic mosaicism and although the mice are on a random bred genetic background, all the X chromosomes were recently derived from a single progenitor and thus should be identical.

X monosomic mice (39,XO) have a remarkably mild phenotype when compared to women with Turner Syndrome (45,XO). In fact, while XO mice are fertile and do not display major physiological abnormalities, TS females present ovarian dysgenesis and other anatomical and physiological abnormalities [25]. A spectrum of neuropsychological deficits is also associated to X chromosome monosomy in humans and, to some extent, in the mouse [24-27]. The main candidates for the TS phenotype are those genes escaping X inactivation in humans but not in the mouse. However, the global degree of escape on the mouse X chromosome is presently unknown.

We present a global expression analysis in somatic tissues of XX and XO mice, using a microarray platform that allows a genome-wide survey of gene expression, followed by a detailed qPCR inspection of the candidate genes. Our analysis identified new genes potentially escaping X inactivation in the mouse and sheds some light on the transcriptional networks regulated by X-linked genes which are disturbed in X chromosome monosomy.

## Results

We performed a genome-wide expression profiling in four different tissues of adult XX and XO mice (brain, liver, kidney and muscle), using the Illumina Mouse WG-6 v1_1 Expression BeadChip, which covers more than 45,000 transcripts and includes 1,326 probes interrogating the X chromosome transcriptome. According to our manual annotation these correspond to 970 different genes, thus achieving an extensive coverage of the mouse X chromosome - on the latest Ensembl release (55, July 2009), approximately 1000 protein coding genes were annotated on the X chromosome. Brain was the tissue where the largest number of probes was detected (24,873 were called present in at least one of the samples), while 19,887 probes were detected in liver, 23,427 in kidney and in muscle 21,820. High values were obtained for the correlation coefficients (r) between biological replicates (0.98 to 1), indicating that the platform and methods applied are extremely robust. This high reproducibility is also likely due to the pooling of individuals of the same genotype (we analyzed 3 pools of at least two individuals for each genotype), which minimizes the differences between individuals. After normalisation the samples were hierarchically clustered according to their global expression profiles, to verify the biological accuracy of the data. The samples clustered primarily by tissue and in general also by genotype, as expected (results not shown).

In the chosen array platform 19,100 probes represent unique curated genes in the NCBI RefSeq database (Build 36, Release 22). We annotated all the remaining probes, by performing automated blast searches followed by a manual curation using combined information from several databases - NCBI http://www.ncbi.nlm.nih.gov, MGI http://www.informatics.jax.org/ and SOURCE http://smd.stanford.edu/cgi-bin/source/sourceSearch. This allowed us to determine the total number of X-linked transcripts expressed in each tissue and to perform a targeted analysis for each dataset. On the X chromosome the highest number of probes was detected in brain (725 present in at least one of the pools), while in liver only 538 were detected; 643 were expressed in kidney and 605 in muscle.

**Figure 1:**
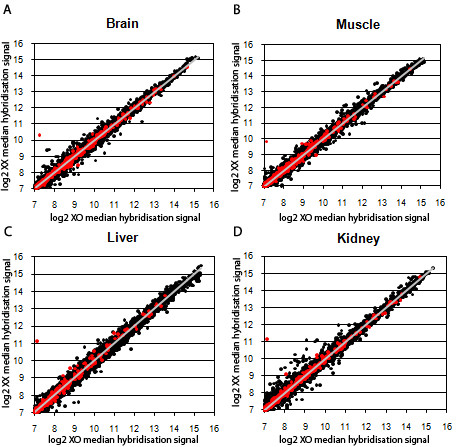
**Plots of the normalised hybridisation signals of XO *versus *XX mice in four tissues**. In black are represented autosomal probes and X-linked genes are in red; the diagonal (in grey) lies at the intersection of points where median expression is equal between groups. The most noticeable X-linked outlier corresponds to *Xist*. A- Brain, B- Muscle, C- Liver, D- Kidney.

As a preliminary analysis of autosomal and X-linked expression in the two groups we plotted the median of the normalised hybridization signals of the XO mice against the XX in each of the four tissues analyzed (Figure 1). In these graphs, the majority of points, representing both autosomal and X chromosome genes, lie within a diagonal where expression is equivalent between groups, indicating that the majority of genes are expressed at similar levels in the two groups. An obvious outlier is present in all graphs, representing *Xist*, an expected observation since this gene is only transcribed from the inactive X chromosome in XX females; a few scattered X-linked genes can be detected which also lie above the diagonal, particularly in liver.

### Differential gene expression on the X chromosome

Recently, several statistical approaches to detect differential gene expression from microarray data have been compared [28] and it was established that empirical Bayes methods perform better than standard t-tests, particularly when the sample size is small. Therefore, to test if those genes located on the X chromosome were differentially expressed between the two groups of mice, we employed a pairwise comparison using an Empirical Bayesian approach, limma [29], which fits a linear model of gene expression to the data. This is a moderate version of the t-statistic where the gene-specific variance estimator takes into account the data from all genes [30]. This variance stabilising approach is very robust to between-sample variation. The resulting p-values were corrected for multiple tests by setting the FDR (False Discovery Rate) to 0.05 and number of comparisons equal to the total number of X chromosome probes detected in each tissue. Finally we tested several candidates by quantitative real time PCR in different tissues. The probes significantly overexpressed in XX mice are listed in Table 1, where the respective p-value obtained from the array data after FDR correction is also indicated. Fold-change (FC) estimates obtained with both techniques are also included for reference, although these should be regarded with caution, since they do not take into account the variation of the samples.

**Table 1:** Probes overexpressed in XX *versus *XO mice in each tissue

		Liver			Kidney			Muscle			Brain		
			**FC**			**FC**			**FC**			**FC**	
**Probe ID**	**Gene**	***P-value***	**Array**	**qPCR**	***P-value***	**Array**	**qPCR**	***P-value***	**Array**	**qPCR**	***P-value***	**Array**	**qPCR**
2649129	***Eif2s3x***	**6,17E-07**	**1,79**	**1,54±0,21**	**1,81E-03**	**1,48**	1,43±0,02	**1,11E-02**	**1,35**	**1,83±0,26**	>0,05	1,38	**1,79±0,33**
2447456	***Utx***	**4,79E-05**	**1,34**	**1,97±0,35**^a^	**2,16E-03**	**1,37**	**1,88±0,15**^c^	**1,53E-03**	**1,60**	**2,04±0,24**^d^	>0,05	1,12	**1,38±0,14**^e^
2506528	***Utx***	**4,96E-05**	**1,62**	**1,97±0,35**^a^	**1,98E-04**	**1,51**	**1,88±0,15**^c^	**4,83E-03**	**1,46**	**2,04±0,24**^d^	>0,05	1,17	**1,38±0,14**^e^
2508945	*Lamp2*	**1,69E-04**	**1,29**	1,04±0,10	>0,05	1,03	-	>0,05	1,02	-	>0,05	1,08	-
2700885	*Rgn*	**8,07E-04**	**1,38**	-	ND	ND	ND	ND	ND	ND	ND	ND	ND
2710166	***Ddx3x***	**9,17E-04**	**1,49**	**1,44±0,25**	>0,05	1,27	1,08±0,10	>0,05	1,09	**1,33±0,10**	>0,05	1,08	**1,56±0,24**
1235933	***Utx***	**2,25E-03**	**1,18**	**1,97±0,35**^a^	**3,35E-03**	**1,18**	**1,88±0,15**^c^	>0,05	1,13	**2,04±0,24**^d^	>0,05	1,04	**1,38±0,14**^e^
1231201	*Pja1*	**2,30E-03**	**1,22**	1,24±0,26	>0,05	1,08	1,33±0,08	>0,05	1	-	>0,05	0,98	-
2473374	*Zdhhc9*	**2,47E-03**	**1,18**	-	>0,05	1,01	-	>0,05	0,94	-	>0,05	1,02	-
1249467	*Rap2c*	**3,13E-03**	**1,45**	-	>0,05	1,18	-	>0,05	0,89	-	>0,05	1,06	-
1220409	*Cetn2*	**3,18E-03**	**1,17**	-	ND	ND	ND	>0,05	0,88	-	>0,05	1,05	
2605212	*Slc6a8*	**3,27E-03**	**1,25**	-	>0,05	0,95	-	>0,05	0,97	-	>0,05	0,97	-
2494747	*Huwe1*	**3,51E-03**	**1,31**	-	>0,05	1,11	-	>0,05	0,96	-	>0,05	0,93	-
2540573	*LOC665281*	**4,61E-03**	**1,34**	-	>0,05	1,05	-	>0,05	0,93	-	>0,05	0,97	-
1247078	*Ogt*	**7,74E-03**	**1,11**	1,12±0,14^b^	>0,05	1,05	-	>0,05	0,94	-	>0,05	0,95	-
1236901	*Maob*	**8,04E-03**	**1,17**	1,04±0,06	>0,05	1,02	-	>0,05	0,97	-	>0,05	1	-
1225930	*LOC100039346*	**8,16E-03**	**1,28**	-	>0,05	1,06	-	>0,05	0,86	-	>0,05	0,88	-
1253604	***Jarid1c***	**8,93E-03**	**1,12**	**1,53±0,06**	>0,05	1,13	1,32±0,19	>0,05	1,12	**1,43±0,01**	>0,05	1,06	1,16±0,14
2740445	*Nsbp1*	**9,76E-03**	**1,31**	0,97±0,02	>0,05	0,98	-	>0,05	0,85	-	>0,05	1,06	-
1251370	*Ndufa1*	**9,78E-03**	**1,15**	1,13±0,30	>0,05	0,84	-	>0,05	0,83	-	>0,05	1,02	-
2694999	***2610029G23Rik***	**9,88E-03**	**1,19**	1,24±0,12	**2,53E-03**	**1,26**	**1,45±0,24**	**4,88E-04**	**1,44**	**1,27±0,14**	>0,05	1,27	1,21±0,10
2639642	*Arhgef6*	**9,90E-03**	**1,12**	-	>0,05	1,04	-	>0,05	1,03	-	>0,05	0,81	-
2751935	*Sytl4*	**1,19E-02**	**1,13**	-	>0,05	1,09	-	>0,05	1	-	>0,05	0,93	-
2623216	*Pgrmc1*	**1,29E-02**	**1,41**	1±0,21	>0,05	1,11	-	>0,05	0,83	-	>0,05	0,87	-
2651886	*Pgk1*	**2,32E-02**	**1,19**	-	>0,05	1,01	-	>0,05	0,96	-	>0,05	1,02	-
2590998	*Acsl4*	**2,38E-02**	**1,21**	-	>0,05	0,95	-	>0,05	1	-	>0,05	0,98	-
1236524	*Msn*	**2,77E-02**	**1,15**	-	>0,05	0,93	-	>0,05	1	-	>0,05	0,98	-
2611676	*Ogt*	**2,82E-02**	**1,16**	1,12±0,14^b^	>0,05	1,1	-	>0,05	0,98	-	>0,05	0,85	-
2637249	*Bgn*	**2,85E-02**	**1,14**	-	>0,05	0,95	-	>0,05	0,94	-	>0,05	0,99	-
1212894	*Cask*	**3,09E-02**	**1,15**	-	>0,05	1,03	-	>0,05	0,98	-	>0,05	0,99	-
2501489	*Was*	**3,19E-02**	**1,10**	-	>0,05	1,05	-	>0,05	0,97	-	>0,05	1	-
1220398	*Armcx2*	**3,20E-02**	**1,17**	-	>0,05	1,09	-	>0,05	1,04	-	>0,05	0,97	-
1229474	*Gm732*	**4,42E-02**	**1,10**	-	>0,05	1,02	-	>0,05	0,99	-	>0,05	0,95	-
2423331	*Rbmx*	**4,54E-02**	**1,09**	-	>0,05	0,99	-	>0,05	1	-	>0,05	0,98	-
1246006	*Armcx4*	>0,05	0,98	-	>0,05	0,98	-	**1,84E-02**	1,30	-	>0,05	1,05	-
1244364	***Pnck***	ND	ND	ND	ND	ND	ND	**1,88E-02**	**1,15**	**1,22 ± 0,27**	>0,05	0,76	-

Indicated are the probes (Illumina ID) which were significantly different in each tissue in the pairwise comparison of XX and XO mice, where only the X chromosome probes were considered. P-values were derived from limma followed by FDR correction. For selected genes, fold changes obtained by qPCR (mean of two experiments ± standard error) are also indicated (^a,b,c,d,e ^- each of these values refers to a measurement using one pair of primers). In bold are the genes for which significant p-values were obtained by both methods in at least one tissue. *Xist *was not included but was significant in all comparisons. ND, not detected; qPCR, quantitative real-time PCR; FC, fold-change.

At the 0.05 significance level (adjusted p-value) different groups of probes were found to be significant in each tissue; *Xist *was the only probe significantly different between the two groups of mice in all tissues (*P *< 0.0001).

*Eif2s3x*, which is ubiquitously expressed and known to escape X-inactivation in both human and mice [11] is more highly expressed in XX mice in all tissues analyzed, although in brain the difference did not reach significance by microarray. Two of the three probes present in the array representing *Utx*, one other well known escapee [10,12], were significantly different between groups in our analysis, presenting a FC higher than 1.3 in all tissues except brain and a third probe was significant in liver and kidney. Although in brain none of the probes for this gene was significant, by real time PCR the corresponding transcript was detected more abundantly in XX mice (FC = 1.38 ± 0.14; *P *< 0.05). *Jarid1c*, also shown to escape X inactivation in the mouse [8], presented a modest fold change in all tissues, reaching significance in liver by microarray (FC = 1.12; *P *< 0.01) and qPCR (FC = 1.53 ± 0.06; *P *< 0.05), and in muscle only by qPCR (1.43 ± 0.01; *P *< 0.05). In kidney and brain the difference in *Jarid1c *expression between the two groups was not significant.

Only a small number of genes were differentially expressed between the two genotypes in muscle and kidney and in brain none of the probes reached the significance threshold. However, the established escapees *Eif2s3x *and *Utx *were significantly overexpressed in the brain of XX females by qPCR, as well as *Ddx3x *(formerly *Dbx*), a potential escapee that had been found to be more highly expressed in female compared to male brain by Northern Blot [19]. In our microarray analysis *Ddx3x *was significantly more abundant in XX females in liver but it did not reach significance in any of the other tissues tested.

In liver a higher number of probes, corresponding to 31 different genes, were significantly overexpressed in XX mice (Table 1); *Eif2s3x*, *Utx, Ddx3x *and *Jarid1c *were all highly significant (*P *≤ 0.01). The fold change differences in expression between the two groups in this tissue ranged from 1.1 to 1.8 for the probes with significant p-values although some were expressed at low levels in both groups (*Ogt*, *Cetn2*, *Arhgef6*, *Jarid1c*, *Slc6a8*, *Sytl4, Was *and *Gm732*). The limma analysis we performed also produces a B-statistic, the log-odds that a gene is differentially expressed, which provides an additional measure of the strength of each candidate. The 11 probes presenting the largest p-values in liver all present a B < 0 and have therefore less than 50% chance of being differentially expressed. *Pgk1*, a gene that undergoes X inactivation, is amongst these, although presenting a small FC. We tested one other gene with B < 0 by qPCR (*Pgrmc1*) but could not confirm differential expression (FC= 1 ± 0.21; P > 0.05), advising caution in considering these 11 genes as differentially expressed.

The array also includes a probe annotated as representing *Sts *(*Steroid Sulfatase*), a gene in the mouse pseudoautosomal region. Keitges *et al*. [31] have suggested that this gene escapes inactivation, based on the comparison of the level of enzyme activity in somatic tissues of XX, XO and XY mice. There are, however other reports with conflicting results in the literature [31-33]. According to our microarray analysis *Sts *is not differentially expressed in any of the tissues tested and we were unable to design appropriate primers on exon-exon boundaries in order to validate our microarray results by quantitative PCR, since the genomic sequence of *Sts *was not available in any of the databases searched (NCBI, UCSC Genome Browser and Ensembl). A Blat search http://genome.ucsc.edu/cgi-bin/hgBlat?command=start of the probe sequence retrieved no matches, indicating that the gene is not on the current genome assembly (July 2007). *Enox *(*AK137473*), another gene recently described as escaping X inactivation [34] is not represented on the array.

We analysed several of the significant genes by both microarrays and qPCR. At present, a standard definition of validation of microarray results by qPCR is still lacking and it is common practice to simply compare FC values. We required significant p-values in both methodologies as criteria for full concordance. This is especially important when small expression differences between groups are sought and it is difficult to define a fold change cut-off value. Five of the genes that were significant by microarray analysis were tested by qPCR in all four tissues (*Eif2s3x, Utx, Ddx3x, Jarid1c *and *2610029G23Rik*). Overall, FC estimates obtained by both methods were roughly concordant and all genes were significantly overexpressed by qPCR in at least one of the tissues tested (see Table 1). *Pja1 *was significant by microarray in liver but by qPCR the differences did not reach significance in liver or kidney, the two tissues where the expression levels allowed the determination of reliable standard curves, although the FC estimates obtained were similar. We could not confirm *Lamp2 *and *Nsbp1 *overexpression in liver by qPCR (1.04 ± 0.10; P < 0.05) and therefore these genes were not tested in the other tissues. Additionally, four of the significant genes presenting FC < 1.2 were tested (*Ogt, Maob, Ndufa1 *and *Pnck*); by qPCR only *Pnck *was significantly overexpressed (FC = 1.22 ± 0.27; P < 0.05).

Amongst the transcripts that reached the significance level in more than one tissue by both approaches, one has not been previously described as escaping X inactivation (*2610029G23Rik*). The function of *2610029G23Rik *and its human homologue *CXorf26 *have not yet been determined.

### Analysis of allelic expression

To definitely prove escape from X inactivation, expression from both the active and the inactive X chromosomes should be demonstrated. Ideally single cell analysis should be performed, since every female is a mosaic of cells with either the maternal or the paternal X chromosome active and therefore in a tissue homogenate of a heterozygous female, expression from both alleles would be detected not only from those genes escaping X inactivation but also from inactive genes. Alternatively a system in which X inactivation is skewed towards one of the X chromosomes can be used, such as 40,T(X;16)16H female mice carrying the Searle's translocation. In these females the normal X chromosome is always inactive [35] and therefore biallelic expression, detected through the analysis of sequence polymorphisms, indicates escape from X inactivation. We have analysed the transcripts of three of the genes detected as differentially expressed in our microarray analysis in cDNA samples of the F1 females of a cross between a T(X;16)16H female, kept on a mixed C3H/He and 129/Sv background, and a FVB/NHsd male. Two of the F1 females from this cross presented DNA sequence polymorphisms within the coding region and/or the UTRs of *Huwe1*, *Arhgef6 *and *Nsbp1*; however, for all three genes tested, at the cDNA level only the allele derived from the active X chromosome was detected by direct sequencing and single-base primer-extension (SNaPshot) (Table 2). The second method is also based in a fluorescent dideoxy terminator reaction and has been applied previously to the detection of allele-specific expression on the X chromosome [7]. We were therefore unable to demonstrate expression from both X chromosomes, for these candidate genes.

**Table 2:** Allelic expression analysis in T16H mice

		Female 1		Female 2	
		**gDNA**	**cDNA**	**gDNA**	**cDNA**
***Huwe1***	rs29297624	CT	C	CT	C
	rs29296351	CG	G	CG	G
	rs29294894*	GT	G	GT	G
	rs29295859	CT	C	CT	C
***Arhgef6***	rs13475263*	AC	A	AC	A
***Nsbp1***	rs29091513*	CT	C	CT	C

Allelic expression of X chromosome genes in two F1 females, from a cross of a 40,T(X;16)16H female and an FVB/NHsd male, detected in genomic DNA (gDNA) and in cDNA from different tissues (liver, kidney, muscle and brain). All SNPs in the table were analysed by direct sequencing in liver; those marked as * were also verified by SNaPshot in liver, kidney, muscle and brain. In all cases only the allele on the X chromosome carrying the translocation (active) was detected in the cDNA.

### Secondary transcriptional alterations

In view of the larger number of X-linked probes found to be more highly expressed in the liver of XX mice, we performed a second analysis including all probes in the array which were detected in this tissue, to verify if any autosomal transcripts were deregulated between the two groups of mice. In this genome-wide analysis 1402 probes (out of 19,887) were significantly different between genotypes at the 0.05 significance level after correcting for the number of genes tested (see Additional file 1); of these, 686 (34 annotated to the X-linked and 584 to the autosomes) were detected more abundantly in XX mice, while the remaining 716 (14 annotated to the X chromosome and 673 to the autosomes) were more highly expressed in the XO genotype. We confirmed by qPCR one of the autosomal genes significantly overexpressed in XX females in liver, *Argininosuccinate synthetase 1*, *Ass1 *(FC = 1.49 ± 0.27; P < 0.05), which codes for one of the enzymes of the arginine biosynthetic pathway. Interestingly, the autosomal genes *Eif2b2 *and *Eif2b5*, which encode two of the subunits of the eukaryotic translation initiation factor 2B, were amongst the most significant probes in this genome-wide comparison (*P *= 0.005 and *P *= 0.007, respectively). In humans, mutations in these genes have been detected in females with POF (Premature Ovarian Failure) and it has been hypothesized that EIF2B2 dysfunction in humans may be related to increased apoptosis of ovarian follicles [36].

To further clarify if the alterations observed in autosomal gene expression were specific to liver, and are thus more likely downstream effects of the differential expression detected for several X-linked genes in this tissue, we extended the genome-wide statistical analysis to the remainder of the tissues. In kidney and muscle datasets only a small number of genes were detected as differentially expressed at a 0.05 significance level after FDR correction (6 genes in kidney: *Clic6, 2510022D24Rik, Erdr, 11810009N02Rik, LOC100047358, Rtn4*; 3 genes in muscle: *Kremen, Casq1, Rab33b*). Therefore the autosomal expression perturbations were more pervasive in the liver of monosomic mice, where the differences in X-linked expression are more pronounced.

#### Enrichment of functional categories

We performed a functional enrichment analysis for the liver dataset in FatiGO http://www.babelomics.org/, by comparing the Gene Ontology (GO) terms corresponding to the significant genes detected at the genome-wide level (1340 annotated genes after removing duplicates; Additional file 1) to the list of all probes present in the array (14,964 annotated probes after removing duplicates), by means of a Fisher's exact test.

Several terms related to metabolic and biosynthetic pathways, namely energy metabolism, were overrepresented in the set of significant genes and cytoplasmic/mitochondrial subcellular location was also more frequent (Table 3). Several of the genes coding for NADH dehydrogenase subunits, the first enzyme (complex I) of the mitochondrial electron transport chain, are differentially expressed between XX and XO mice, although there is no clear trend towards an up- or down-regulation of the autosomal components of this enzymatic complex. *Ndufa1*, the subunit encoded by an X-linked gene, is significantly overexpressed in XX females by microarray but not by qPCR. The modest increase in expression of this gene, as well as the differences found in other energy metabolism genes, such as *Cytochromeb5 Reductase1 *(*Cyb5r1*) and the *Crystallin Zeta *quinone reductase (*Cryz*), and in genes encoding other mitochondrial proteins, may be due to a global alteration of the gene expression networks relevant for mitochondrial metabolism.

**Table 3:** GO biological processes overrepresented in the list of deregulated genes in liver

GO group	P-value
cytoplasmic part (GO:0044444)	3,35E-10
cytoplasm (GO:0005737)	2,11E-09
mitochondrion (GO:0005739)	6,64E-07
ribonucleoprotein complex (GO:0030529)	1,23E-04
mitochondrial part (GO:0044429)	1,92E-04
intracellular part (GO:0044424)	1,19E-03
biosynthetic process (GO:0009058)	1,69E-03
organelle envelope (GO:0031967)	1,85E-03
organelle inner membrane (GO:0019866)	2,22E-03
cellular metabolic process (GO:0044237)	4,51E-03
intracellular (GO:0005622)	6,98E-03
membrane-bound organelle (GO:0043227)	7,50E-03
organelle part (GO:0044422)	7,50E-03
ribosome (GO:0005840)	1,24E-02
cellular biosynthetic process (GO:0044249)	1,60E-02
organelle membrane (GO:0031090)	1,65E-02
translation (GO:0006412)	1,66E-02
intracellular membrane-bound organelle (GO:0043231)	2,52E-02
intracellular organelle part (GO:0044446)	2,52E-02
proteasome complex (sensu Eukaryota) (GO:0000502)	4,21E-02
intracellular organelle (GO:0043229)	4,21E-02

Functional groups regulated in liver whole genome analysis. Functional enrichment analysis performed in FatiGO http://www.babelomics.org/ by Fisher's exact test followed by FDR correction considering the number of functional groups tested.

#### Enrichment of transcription factor binding sites

Considering that several functional categories are overrepresented in our liver dataset it is plausible that the expression of some X chromosome genes may be perturbed indirectly through the action of shared transcription factors within an expression network. To further explore this hypothesis we searched for conserved binding sequences for known transcription factors within the 5000 bp upstream and downstream the TSS of those genes that were overexpressed in XX compared to XO females and performed an analysis of TFBS enrichment using the oPOSSUM web tool http://www.cisreg.ca/oPOSSUM/. Following FDR correction the genes overexpressed in liver showed enrichment for six transcription factors (NKX3-1, Lhx3, HLF, Foxa2, Prrx2, Foxd3; P < 0.05, Fisher's exact test).

Interestingly, several overexpressed genes located on the X chromosome, including *Huwe1*, *Arhgef6 *and *Nsbp1*, present TFBS for one or more of these transcription factors. It seems plausible that in XX females higher transcriptional output of some of the genes that escape X inactivation may lead to a secondary upregulation of other genes on the X chromosome belonging to the same transcriptional networks, which will therefore be overexpressed even though not escaping X-inactivation.

## Discussion

In the present analysis we aimed to detect differences in X-linked expression between XX and XO mouse females in four different tissues, to fully characterize the genes escaping X inactivation in this model and to increase our understanding of the basis of the different phenotypes of X monosomic females in humans and mice.

The identification of genes differentially expressed through the statistical analysis of microarray data represents a challenge and the application of this technique to the identification of genes escaping X inactivation is of added difficulty. Due to the biological nature of the differences we were aiming to detect, i.e., expression from the active X chromosome, which is inherently upregulated [1], and from the inactive X, which should reach at most half of that of its counterpart, a modest overexpression of a maximum of 1.5-fold is expected between groups for those genes escaping inactivation [37]. In fact, it has been demonstrated experimentally that expression from the inactive X chromosome, compared to the active X, can be as low as 5%-15% [7]. Therefore, minor differences between XX and XO mice for genes escaping X inactivation are not unexpected.

While there was an overall agreement on the direction of change obtained by microarray and qPCR in our study, the concordance between the two approaches was not complete. It is widely accepted that several factors can contribute to discrepancies between microarray and qPCR data and each technique presents advantages and pitfalls. In a recent assessment it has been established that even though lower fold changes in microarray data (<1.4) are less likely to be confirmed by qPCR, the most determinant factor in obtaining a high correlation for these two approaches is the microarray data p-value [38]. The same trend is supported by our results.

The most consistent results across tissues, obtained by both methods, were those of two of the genes previously demonstrated to escape X inactivation, *Eif2s3x *and *Utx*, and for a new candidate gene (*2610029G23Rik*). *Jarid1c*, also known to escape X inactivation, and *Ddx3x*, a potential escapee, only reached significance by both methods in liver. Variable expression from the inactive X chromosome across tissues has been reported for *Jarid1c *[39,40] and may have a bearing on the results, since very small expression differences may be beyond the detection limit of both approaches.

The majority of the genes detected as differentially expressed were only so in one of the tissues analyzed, with liver presenting a noticeably larger number of genes significantly overexpressed. Several factors may underlie these observations. Variable escape from inactivation has been observed in rodent-human somatic cell hybrids and in human cell lines [7,41] as well as across human [42] and mouse tissues [39,40] and therefore inactivation patterns of some genes may be tissue- or even cell-type specific. Tissue composition is also likely to be contributing to the differences observed, particularly in brain, an organ with very complex tissue architecture, where we and others [43] were unable to detect significant differences between XX and XO mice by microarray, even though a high proportion of genes on the X chromosome are highly expressed in this tissue [1].

For several genes, more than one probe was detected and, in many cases, different probes were not concordant in defining a gene as significantly overexpressed, even in the same tissue. A careful analysis revealed that those probes with discordant results were often derived from genomic sequences that were not included in all transcripts of a given gene. Therefore, the existence of alternative splicing is contributing to some extent to the heterogeneity observed between tissues. Additionally, our analysis of enrichment of functional categories and TFBS within the group of differentially expressed genes suggests that secondary transcriptional regulation has an impact on the expression level of several X chromosome genes and regulation at this level is also likely to vary between tissues.

Even though the global transcriptional output from the X chromosome is similar in males and in females [1], two studies of genome-wide gene expression reported several genes differentially expressed between the sexes in the mouse [16,17]. Notably, Yang and collaborators [17] analyzed a large number of individuals (169 females and 165 males) and found several genes on the X chromosome more highly expressed in females, in at least two tissues (of the four included in the analysis). In this dataset *Utx *was female-biased in all tissues analyzed (brain, liver, adipose and muscle) and *Eif2s3x, Ddx3x *and *2610029G23Rik *were also female-biased in 3 of the four tissues. Only those genes with a minimum of 3-fold differential expression between sexes were reported by Rinn and colleagues [16] but, in a reanalysis of their data, we found that *Eif2s3x *is significantly overexpressed in female hypothalamus; *Utx*, *Ddx3x *and *2610029G23Rik*, amongst others, presented a fold change of 1.2 or higher in at least one of the tissues (kidney, liver and hypothalamus), although not reaching the threshold of significance. The fact that a much larger number of genes is female-biased in at least two tissues (56 genes at *P *< 0.05) in the comparison performed by Yang *et al*. [17] is most likely due to their larger sample size. Although some of these differences may be caused by other factors not directly related to the chromosome complement, such as sex-specific hormonal influences, the fact that several of the genes were consistently overexpressed in both male/female comparisons and in our analysis of XX and XO mice suggests that some may indeed escape X inactivation. In agreement with these results, *Ddx3x *is indeed expressed from the inactive X chromosome in the mouse (L. Carrel, personal communication).

In our study, two new candidates tested were concordant at *P *< 0.05 by both approaches, in at least one of the tissues (*2610029G23Rik *and *Pnck*). Although higher expression of *Pja1 *in the liver and kidney of XX females was also observed by qPCR, the difference did not reach significance by this method. Both *Pnck *and *Pja1 *are female-biased in the adipose tissue according to the data of Yang *et al*.[17]. Additionally, several of the genes that were significant in liver in our microarray analysis but were not confirmed by qPCR (*Ogt, Maob,Ndufa, Pgrmc1 *and *Nsbp1*) are amongst the genes that are female-biased in at least one tissue, according to the data of Yang *et al*. [17]. In the latter study most of the sex differences in gene expression were modest (FC < 1.2) and the authors did not perform qPCR experiments to confirm them.

Further evidence in support of the existence of uncharacterised escapees on the rodent X chromosome comes from studies in mouse ES cell lines [15,44]. However, in the absence of direct evidence of escape from X inactivation from our allele-specific analysis, two alternative hypothesis must be considered: i) the three genes tested (*Huwe1*, *Arhgef6 *and *Nsbp1*) are false positives generated by the statistical analyses of differential expression by microarray (both in our study and in the study of Yang *et al*.); ii) overexpression of these genes in XX females may be due to a secondary transcriptional upregulation of the active X chromosome allele and does not reflect expression from both X chromosomes, as discussed above. The former hypothesis would imply a bias skewing the results in the same direction in two independent studies using different microarray platforms and a different study design, which is unlikely. On the other hand, the fact that these three genes present binding sites for common transcription factors gives strength to the second hypothesis, where overexpression of some X-linked genes would be due to transcriptional upregulation, through the same mechanism that is contributing to higher expression of several autosomal genes in XX females.

Our results, as well as the results from other studies using both *in vitro *and *in vivo *models suggest the existence of tissue- or cell type-specific patterns of X inactivation. However this issue can only be fully addressed by a thorough gene-by-gene comparison of allelic expression in single cell *versus *whole tissue homogenates.

### Relevance for understanding the molecular basis of Turner Syndrome

In humans, complete or partial monosomy of the X chromosome results in Turner syndrome. The phenotype, which includes ovarian dysgenesis and infertility, is attributed to a lower dosage of X chromosome genes escaping inactivation. However, only 5% of the genes on the human X chromosome that are expressed in lymphoblastoid cell lines are significantly overexpressed in females at the population level [37]. Therefore, only a small number of genes are predicted to contribute to dosage imbalances in X chromosome aneuploidies.

We detected lower expression of several X-linked genes in XO mice. Moreover, a large number of autosomal genes were differently expressed between the two genotypes in liver, including two genes which are mutated in POF - *Eif2b2 *and *Eif2b5 *[36]. Due to the high number of genes on the X chromosome involved in ovarian and oocyte development it will be interesting in the future to analyse X-linked expression in the ovary. However the tissue composition of this organ is highly heterogeneous, including both somatic and germinal cell lineages and therefore reactivation of the inactive X chromosome during oogenesis is a confounding factor that must be considered.

The analysis of functional enrichment we performed in the liver dataset revealed other cellular processes that are differently regulated between the two groups of mice analyzed - ATP synthesis and mitochondrial metabolism. Impairment of energy metabolism is expected to lead to several alterations in cell homeostasis and is known to play a prominent role in the pathogenesis of neurological diseases. These results may therefore have a bearing on some of the observed neuropsychological deficits of TS. The presence of binding sites for shared transcription factors within the regulatory regions of the genes underexpressed in XO mice reinforced the hypothesis of a deregulation of specific pathways in this model.

Although X-linked imprinted genes can also contribute to some of the phenotypes observed in TS females, parental effects on expression have so far only been detected in some genes of the *Xlr *family [24,43]. In our analysis no relevant differences were found between X^m^O and X^p^O mice.

## Conclusion

We confirmed and extended previous global analysis of X chromosome gene expression in the mouse and uncovered a dosage-dependent effect on the expression levels of several X-linked and autosomal genes that cannot be attributed to sex-specific hormonal influences. Even though we found strong candidates for escaping X inactivation, the lack of direct evidence of escape for those genes tested suggests that other factors may underlie the differences observed between XX and XO mice.

Several X-linked and autosomal genes are deregulated in XO mice and these are involved in a variety of cellular functions. We hypothesize that the TS phenotype is partially caused by the additive effect of regulatory perturbations downstream of the under-expressed X-linked genes, which may differ between tissues and between species. Moreover, genes escaping inactivation in humans but not in the mouse may lie in regulatory nodes at the intersection of a larger number of effectors, having a greater impact on genome-wide levels of expression.

## Methods

### Subjects

The mouse models used in our expression analysis were obtained as described in [24], except that in our experiment the X chromosomes of the random bred MF1 genetic background derived from a single progenitor X and thus are the same for all individuals analyzed (X^m^O, X^p^O and X^m^X^p^). All genotypes were determined by bone marrow metaphase preparations and in some cases confirmed by PCR. All animal procedures and breeding were in accordance with the United Kingdom Animal Scientific Procedures Act 1986 and were subject to local ethical review.

### RNA extraction

Tissues from 12 week old mice were harvested and stored at -80°C before use. Total RNA was isolated from homogenized tissues using Trizol reagent (Invitrogen) and purified using the RNeasy Kit (Qiagen) according to the manufacturer's instructions. RNA integrity was verified by capillary electrophoresis in the Agilent 2100 Bioanalyzer.

### Gene expression quantification in 40,XX and 39,XO mice

The RNA samples of seven 40,XX, eight 39,X^p^O and eight 39,X^m^O mice were pooled by genotype into 9 groups, representing 3 biological replicates per genotype, as follows: 39,X^p^O-1 and 39,X^p^O-2 (3 pooled individuals each), 39,X^p^O-3 (2 pooled individuals); 39,X^m^O-1 and 39,X^m^O-2 (3 pooled individuals each), 39,X^m^O-3 (2 pooled individuals); 40,XX-1 and 40,XX-2 (3 pooled individuals each) 40,XX-3 (2 pooled individuals). A total of 300 ng of RNA from each pool was then amplified following the Illumina TotalPrep RNA amplification protocol. The samples were labelled and hybridized to the Mouse WG-6 v1_1 Expression BeadChip whole genome expression array available from Illumina, following the manufacturer's protocols.

### Array data extraction, normalisation and analysis

The values for each bead in the array were imported into the Illumina software BeadStudio for preliminary quality checks, summarized according to bead type and then exported into R environment for statistical computing. Normalisation across all arrays was performed on a log scale using the quantile normalisation method [45] implemented in lumi normalisation package [46]. The normalised intensity values were used for independent pairwise comparisons for each tissue, using the empirical Bayes approach implemented in limma by assigning the samples to two groups according to genotype (40,XX and 39,XO). Only probes detected as present in at least one the samples in each tissue were compared. The p-values were then subjected to the FDR correction[47].

Samples were hierarchically clustered using the tools implemented in InforSense Workflow Builder http://www.inforsense.com. The distance matrix was calculated with Pearson correlation and clusters built by average linkage.

The microarray data for this study have been deposited to GEO under accession number GSE13520.

### Quantitative Real-Time PCR

Quantitative real-time PCR reactions were carried out using 100 ng of cDNA in a reaction volume of 20 μl comprising Sybr Green PCR Master Mix (Applied Biosystems) and 0.3 μM of each primer in an Applied Biosystems 7000 detection system (Applied Biosystems). Efficiency of primers and quantity of cDNA in each well were derived from an experimentally determined standard curve; only reactions with r^2 ^≥ 0.99 and with a standard curve slope typically -3.1 ≤ S ≤ -3.6 were accepted. Melt curve data were obtained to confirm amplification of the correct product. Expression was normalised by reference to *Gapdh *and in most cases the results were confirmed using *Actb*. Differences in expression between groups were calculated by means of a t-test. All primers were designed to span exon-exon boundaries, to prevent amplification of possible genomic DNA contaminants. For primer sequences see Additional file 2.

### Analysis of allelic expression

Expression from each X chromosome was verified using polymorphisms within the coding region and/or the UTRs of *Huwe1*, *Arhgef6 *and *Nsbp1 *by direct sequencing and, whenever possible, by single-base primer-extension (SNaPshot). Each SNaPshot assay was tested in genomic DNA of T16H-FVB F1 females heterozygous for the target SNPs and then used to differentiate expression from each X chromosome in the cDNAs from liver, kidney, muscle and brain of two animals. Typically 50 ng of DNA and 100 ng of cDNA were amplified using the HotStar HiFidelity Polymerase Kit (Qiagen) in a 10 μl reaction volume comprising 0.3 μM of each primer. PCR conditions were the following: 15 min pre-incubation step at 94°C, 35 cycles of denaturation at 94°C for 30 sec, annealing for 45 sec at the respective AT for each primer pair and extension at 72°C for 45 sec, followed by a final extension step at 72°C for 10 min. The PCR products were then purified with ExoSAP-IT (Usb) and single-base primer-extension reactions were carried out with SNaPshot Multiplex Kit (Applied Biosystems) in a 5 μl volume comprising 0.2 μM of the extension primer and up to 2 μl of the purified PCR product (depending on the expression level of each gene in the different tissues tested), for 25 cycles. SNaPshot reactions were cleaned up with Shrimp Alkaline Phosphatase (Usb) and the analysis of fluorescent products was performed in an ABI 3100 sequencer using the GeneMapper 4.0 Analysis Software. For primer sequences see Additional file 2.

## Abbreviations

XIC: X Inactivation Center; LTRs: Long Terminal Repeats; ES: Embryonic Stem (cells); TS: Turner Syndrome; FDR: False Discovery Rate; FC: Fold Change; POF: Premature Ovarian Failure; GO: Gene Ontology.

## Authors' contributions

AML conceived the study, designed and carried out all the experiments, analysed the data and wrote the manuscript; PSB and AO bred the mice and verified the genotypes; JB contributed to data analysis; NAA, CAS and PSB contributed to the experimental design, discussion of the results and with a critical review of the manuscript; AA contributed to the discussion of the results and with a critical review of the manuscript. All authors read and approved the final manuscript.

## Supplementary Material

Additional file 1 **Probes differentially expressed in liver of XX and XO mice**. A linear model pairwise comparison between XX and XO mice was performed using the R package limma for all probes (X chromosome and autosomal) detected in liver (19,887). After FDR correction 1402 probes were significantly different between genotypes at the 0.05 significance level. Click here for file

Additional file 2 **Primer sequences**. This file contains the sequences of the primers used in qPCR and in the analysis of allelic expression by SNaPshot. Click here for file
